# 

**Published:** 1858-09

**Authors:** 


					﻿PUBLISHED MONTHLY, AT S3 PER ANNUM IN ADVANCE.
X
©
3
3
©
©
a
e
‘E
l
©
3
©
>»
d
x
s
©
©
co
z
©
©
©
>
©
©
©
>•
d
x
S
©
©
kO
r-l
x
—
a
©
©
fa
©
©
S
P
I
W
5
§
£
JV T L A N T _A_
MEDICAL AND MB
: JO W R N A 1 «.
EDITED BY
JOSEPH P. LOGAN, M. D.,
1
Professor of Physiology and Gexerkl Pathology in the Atlanta Medical College.
a N D
W. F. WSTMORELAND, M. D.,
Professor op the Principles and Practice of Surgery in the Atlanta Medical College,
Pax et scientia, sed veritas sine timore.
Vol. IV. SEPTEMBER, 1858. No. I.
ATLANTA, GEORGIA:
PRINTED BY G- P- EDDY & CO-
18 5 8.
each number contains sixty-four pages.
“S
©
X
1 s
5
X
©
X
p
*1
■	t
, ©
A
s
©
X
i ©
©
• ©
©
1
I
£
■	S-
?
©
p
zr
ft
1 z
*
I x
3
1 1
©
©
£
©
s*
©
©
d
W
X
>
r
X
*1
©
X
©
G.
P
r*
3*
©
©*
©
B
©
©
X
				

